# Rheumatic Fever: A Disease without Color

**DOI:** 10.5935/abc.20190141

**Published:** 2019-09

**Authors:** Estevão Tavares de Figueiredo, Luciana Azevedo, Marcelo Lacerda Rezende, Cristina Garcia Alves

**Affiliations:** 1 Faculdade de Medicina de Ribeirão Preto (FMRP) da Universidade de São Paulo (USP), Ribeirão Preto, SP - Brazil; 2 Universidade Federal de Alfenas, Alfenas, MG - Brazil

**Keywords:** Rheumatic Fever, Rheumatic Heart Disease, Cardiovascular Surgical Procedures/mortality, Hospitalization/economics, Antibiotic Prophylaxys/economics, Public Health Policy

## Abstract

**Background:**

Brazil has approximately 30.000 cases of Acute Rheumatic Fever (ARF) annually. A third of cardiovascular surgeries performed in the country are due to the sequelae of rheumatic heart disease (RHD), which is an important public health problem.

**Objectives:**

to analyze the historical series of mortality rates and disease costs, projecting future trends to offer new data that may justify the need to implement a public health program for RF.

**Methods:**

we performed a cross-sectional study with a time series analysis based on data from the Hospital Information System of Brazil from 1998 to 2016. Simple linear regression models and Holt’s Exponential Smoothing Method were used to model the behavior of the series and to do forecasts. The results of the tests with a value of p < 0.05 were considered statistically significant.

**Results:**

each year, the number of deaths due to RHD increased by an average of 16.94 units and the mortality rate from ARF increased by 215%. There was a 264% increase in hospitalization expenses for RHD and RHD mortality rates increased 42.5% (p-value < 0.05). The estimated mortality rates for ARF and RHD were, respectively, 2.68 and 8.53 for 2019. The estimated cost for RHD in 2019 was US$ 26.715.897,70.

**Conclusions:**

according to the Brazilian reality, the 1-year RHD expenses would be sufficient for secondary prophylaxis (considering a Benzatin Penicillin G dose every 3 weeks) in 22.574 people for 10 years. This study corroborates the need for public health policies aimed at RHD.

## Introduction

According to the Brazilian Institute of Geography and Statistics (IBGE), Brazil has 10 million cases of pharyngotonsillitis every year, leading to approximately 30.000 cases of Acute Rheumatic Fever (ARF).^1^ Rheumatic Heart Disease (RHD) has a low incidence in developed countries, with 0.1 to 0.4 cases/1,000 school children in the US, while in Brazil these values are 7 cases/1.000 school children, showing that it is directly associated with environmental and socioeconomic factors.^2^ Approximately 70% of the patients with acute RF develop carditis and a third of the cardiovascular surgeries performed in Brazil are due to of RHD sequelae.^3,4^ RF was responsible for 5.1 million potential disability-adjusted life years (DALYs), resulting from 280.000 deaths in 2004, and it was the seventh and eighth causes of mortality and morbidity due to neglected diseases, respectively.^5^

Rheumatic fever is a disease with a cross-linked autoimmune nature triggered by susceptible host response after pharyngotonsillitis by Group A β-hemolytic *Streptococcus.*^6-8^ The implementation of treatment for pharyngotonsillitis by Group A β-hemolytic *Streptococcus* with Benzathine Penicillin G (BPG) within nine days of symptom onset can eradicate the infection and prevent a first outbreak of acute RF^3^ or a new outbreak,^9^ which was already advocated by the WHO in 1955.^10^ Unfortunately, the expected infection eradication rates do not seem to have been reached in Brazil, as shown by our analyses of data from the Health Information System (SIH) from the Brazilian National Health System (SUS).^11^

SUS guarantees universal and egalitarian access to health care and services to everyone in the national territory. Therefore, Brazil’s health policies include care by the public (SUS) and the private sectors (supplementary healthcare, or private health plans), plus care by the private sector within the public sector (complementary health) and by the public sector within the private sector (regulation, inspection, surveillance). Herein, we disclose the cost analysis of health care and services related to RF and RHD incurred by SUS, i.e. under public management, which is different from that of the private systems.

Considering the presented data and the absence of a national RF and RHD prevention program, the objective of this study was to analyze the historical series of mortality rates and disease costs, projecting future trends to offer new data that may justify the need to implement a public health program for RF. In addition, we estimate the annual costs of the diseases and their comorbidities in Brazil. Moreover, the RHD mortality rate was compared with breast (BC) and prostate (PC) cancer mortality rates, which already have implemented public health programs, such as the case of the Pink October^12^ and the Blue November,^13^ respectively.

## Methods

A cross-sectional ecological study with a time series analysis was developed to analyze the historical series of mortality rates and disease, using data from the SIH/SUS^11^ from 1998 to 2016. The year 2017 was not included in this study because the data were still subject to updates.

To estimate the annual cost of the diseases and their comorbidities in Brazil, first we determined the costs associated to the diagnosis of ARF, primary and secondary prophylaxis of RHD, as well as public expenses associated to the consequences of RHD, such as interventional procedures and hospitalizations for heart failure, atrial fibrillation, ischemic stroke and infective endocarditis. For this purpose, the data were obtained as follows: the procedures required for the diagnosis of ARF and Jones Criteria, which have been reviewed at irregular intervals by the American Heart Association (AHA), adjusted for RHD. For hospitalization costs, we considered the mean length of hospital stay of 7 days for ischemic stroke, heart failure 4 days, 4 days for atrial fibrillation and 17 days for infective endocarditis.^14^ The data related to cost of the procedures required for the diagnosis of ARF/RHD and hospitalizations due to consequences of RHD were taken from the database of the Table Management System of Procedures, Medical drugs, Orthotics, Prosthetics and Special Materials of SUS (SIGTAP)^14^ and the Drug Market Regulation Chamber (CMED) of the National Agency of Sanitary Surveillance (ANVISA).^15^ These data are available at the Hospital Information Systems - SIH/SUS -Brazilian Health System. Second, we developed a hypothetical scenario based on the current panorama of rheumatic fever in Brazil, crossing data from the Brazilian Institute of Geography and Statistics with data from the REMEDY study^16^ with their respective morbidities in numbers, to estimate the number of cases. The REMEDY study involved 25 sites in 12 African countries, Yemen and India. Countries were grouped into three income categories: low-income countries (Ethiopia, Kenya, Malawi, Rwanda, Uganda and Zambia), low-middle income countries (Egypt, India, Mozambique, Nigeria, Sudan and Yemen) and middle-income countries (Namibia and South Africa).^16^ The costs obtained were multiplied by the number of cases of group A Streptococcus (GAS) infection, ARF, RHD, and RHD morbidity.

Moreover, the RHD mortality rate was compared with breast (BC) and prostate (PC) cancer mortality rates, which was performed taking in account the period of 18 years (1998 to 2016), using data from the Mortality Information System's (SIM) of SUS - DATASUS,^11^ responsible for the maintenance of mortality data in Brazil. For this comparison, a simple linear regression was adjusted to each case (RHD, PC, and BC).

The present study used only secondary data obtained from public access sources. The approval of this study was waived by the Research Ethics Committee, as established in Resolution 510 of the National Health Council (CNS) of April 7, 2016.

### Statistical analyses

To evaluate the trend of the historical series, simple linear regression models were adjusted. When working with time series, it is common to find problems of heteroscedasticity and autocorrelation. In order to deal with these problems and to allow the performance of valid inferences for the adjusted models, as well as to guarantee the robustness of the models, the HAC (Heteroskedasticity and Autocorrelation Consistent) was used for the covariance matrix of the estimated coefficients.^17^ To model the behavior of the series and make predictions, Holt's Exponential Smoothing Method was used.^18^ R software (version 3.2.4) was used for the statistical analysis. The results of the tests with a value of p < 0.05 were considered statistically significant. The limitation of this study was the analysis of the SIH (SUS) database,^11^ of which data are entered every two months or more, limiting confidence only to total annual data.

## Results

**Figure 1:**
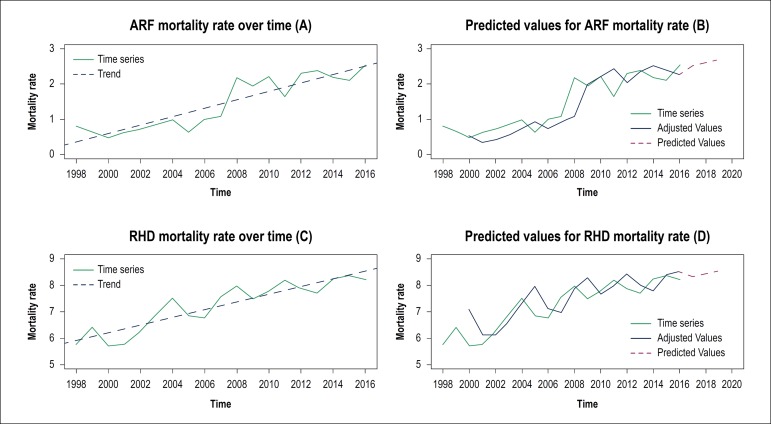
Growth trends and predicted values for Acute Rheumatic Fever (ARF) and Rheumatic Heart Disease (RHD) mortality rates. The model equation for the trend of the ARF (A) mortality rate was ARF_MT_ = –237,79 + 0,12* Year, whereas for the trend of the RHD mortality rate (C) it was RDH_MT_ = –286,11 + 0,15* Year. It should be noted that all trends were significant (p-value < 0.050), evidencing the increasing trend of the series over time.

Mortality rates from Acute Rheumatic Fever (ARF) and Rheumatic Heart Disease (RHD) showed an increasing pattern throughout the analysis period (Figure 1). The ARF mortality rate increased from 0.80 in 1998 to 2.52 in 2016, a growth of 215%, with an increase of 0.12 units, on average, with each passing year (Figure 1A). The RHD mortality rate was 5.77 in 1998, increasing to 8.22 in 2016 (a growth of 42.5%), showing an average rate increase of 0.15 units per year (Figure 1C). Using Holt’s Exponential Smoothing, it was possible to perform mortality estimates for ARF and RHD. The predicted values for ARF mortality rate for 2018 and 2019 were, respectively, 2.59 and 2.68, while the predicted values for RHD mortality rates were 8.43 for 2018 and 8.53 for 2019.

Although these numbers may be underestimated de to the lack of a health surveillance strategy, which will be discussed later, 732 deaths were recorded in 2003 and after a linear regression (p-value < 0.005) of the entire studied period, it is observed that the number of deaths increases on average 16,94 units each year.

Regarding the cost analyses, Table 1 shows a detailed description of the obtained costs for ARF diagnosis, the most common interventional procedures in RHD, and the costs of hospitalization due to the consequences of RHD, for a hypothetical patient in the context of the Brazilian public health system.

**Table 1:** A detailed description of the costs associated with the diagnosis of Acute Rheumatic Fever, as well as the costs of interventional procedures and hospitalizations due to Rheumatic Heart Disease in the context of the public health system of Brazil (values set for 2016)

Diagnosis and treatment	Procedures	Individual cost per procedure	
		(R$)	(US$)^†^
Procedures needed for the diagnosis of ARF*	Medical consultation	10.00	3.04
	Electrocardiogram	5.15	1.56
	CRP	2.83	0.86
	ESR	2.73	0.83
	ASO	2.83	0.86
	Oropharyngeal Culture	5.72	1.74
	Transthoracic echocardiogram	39.64	12.03
	Rapid antigen detection test for GAS	4.33	1.31
Interventional procedures in RHD*	Valve repair	6,061.70	1,840.33
	Valve replacement	6,321.74	1,919.28
	Multiple valve replacement	7,277.56	2,194.28
	Percutaneous mitral valvuloplasty	1,739.19	528.01
Hospitalization due to the consequences of RHD*	Ischemic stroke	1,635.55	496.55
	Heart failure	699.46	212.35
	Atrial fibrillation	219.65	66.68
	Infective endocarditis	880.00	267.68

ARF:acute rheumatic fever; RHD: rheumatic heart disease; CRP: c-reactive protein; ESR: erythrocyte sedimentation rate; ASO: antistreptolysin O; GAS: group A β-hemolytic Streptococcus.

* Data from Jones Criteria, reviewed by the AHA.

† The values in US dollars (US$) were obtained on February 8, 2018. One Brazilian Real (R$) was equivalent to US$ 0.3036. Source: The authors.

**Figure 2:**
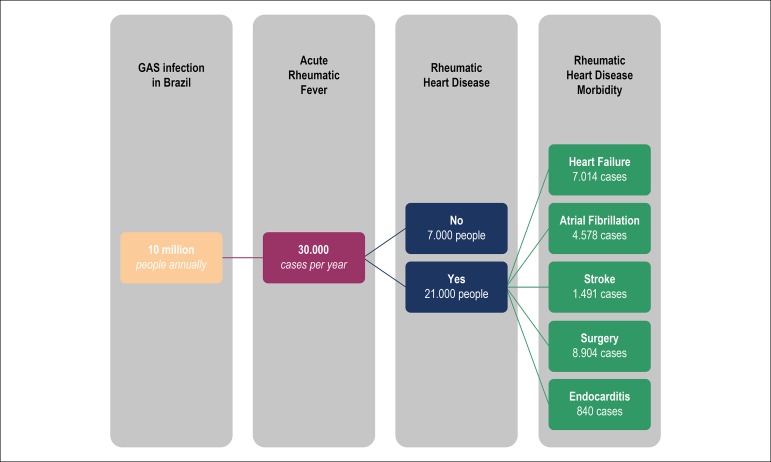
GAS: Group A β-hemolytic Streptococcus. Hypothetical scenario based on the current panorama of rheumatic fever in Brazil, after crossing data from the Brazilian Institute of Geography and Statistics with data from the REMEDY study,16 showing the evolution of Acute Rheumatic Fever to Rheumatic Heart Disease, with their respective morbidities in numbers.

With an average of 30,000 ARF cases per year in Brazil, in a hypothetical scenario based on the REMEDY study,^16^ we would have the scenario shown in Figure 2. According to this hypothesis, there would be 21.000 cases of RHD per year, which would lead to approximately 7.014 new patients with heart failure, 4.578 cases of atrial fibrillation, 1.491 cases of stroke, 8.904 cardiac surgeries and 840 cases of infective endocarditis.

**Figure 3:**
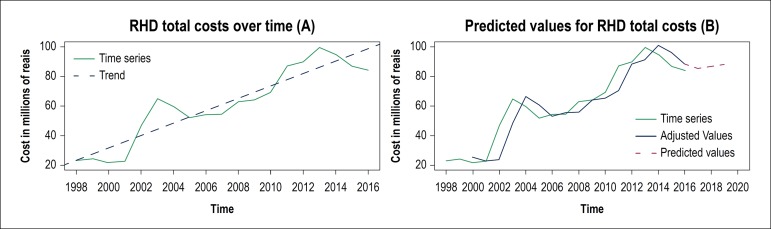
Growth trends (A) and predicted values (B) for total costs with RHD. The model equation for the total costs with RHD (C) was RDH_TC_ = –8346,31 + 4,19* Year. It should be noted that all trends were significant (p-value < 0.050), evidencing the increasing trend of the series over time.

As shown in Figure 3A, total expenditures with hospitalization in Brazil for RHD increased by 264% in the analyzed period, from R$ 23.077.356,65 (US$ 7.006.288,21) in 1998 to R$ 84.080.772,39 (US$ 25.526.924,01) in 2016. The costs in the analyzed period were recorded in 2013 (R$ 99.476.203,42 or US$ 30.200.975,35). Therefore, applying Holt’s Exponential Smoothing method, the predicted values for total costs related to RHD (Figure 3B) were R$ 86.691.610,00 (US$ 26.319.572,79) and R$ 87.997.028,00 (US$ 26.715.897,70) for 2018 and 2019, respectively.

**Figure 4:**
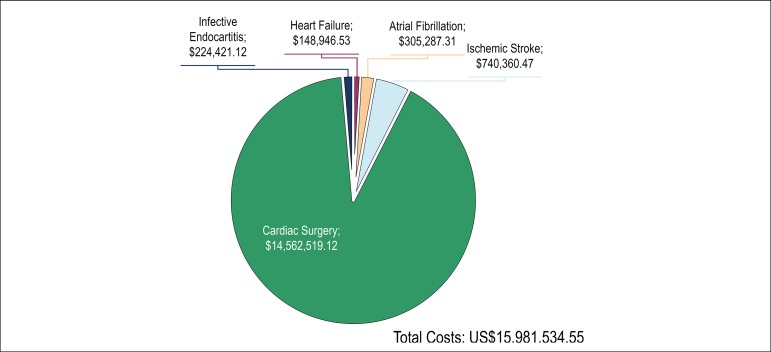
Projection of estimated minimum annual costs in US dollars for Rheumatic Heart Disease morbidities. The final values were calculated based on the case estimates made in Figure 2, multiplying by the values detailed in Table 1, taking into account only one procedure or one hospitalization for each patient over time.

Considering this hypothetic scenario, where all the morbidities required at least one hospitalization and the regular values of cardiac surgeries, the expenses for the Brazilian public health system would have a minimum annual cost of R$ 56.726.131,10 (US$ 15.981.534,55), as shown in Figure 4.

**Figure 5:**
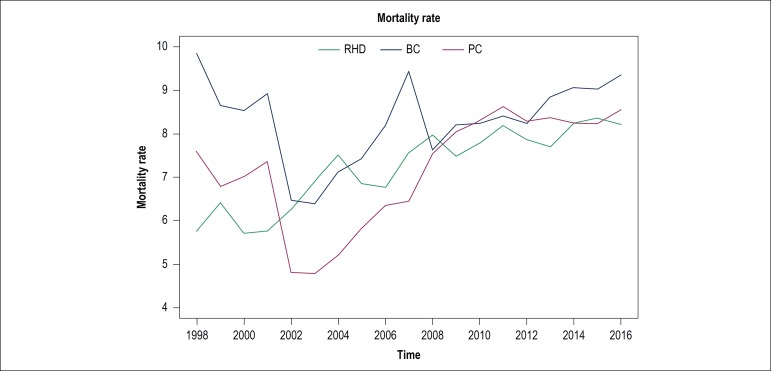
Comparison between the increase of mortality rates for Rheumatic Heart Disease (RHD), Prostate Cancer (PC) and Breast Cancer (BC). According to the adjustment of a simple linear regression for each of the series, the trends for RDH (0.15 [0.12, 0.17]) and BC (0.14 [0.07, 0.22]) were significant (p-value < 0.050) and did not show any significant difference, as the confidence intervals overlapped. The trend for PC (0.04 [-0.04; 0.12]) was not significant (p-value > 0.050).

Taking as reference the mortality rates from two diseases with a high global prevalence, breast cancer and prostate cancer, of which magnitude generated the preventive task force established by worldwide campaigns (Pink October and Blue November), RHD mortality behaves in a similar manner (Figure 5). In this sense, we highlight that growth trends of RHD and BC are significant; however, there are no significant differences between them, which is demonstrated by the overlap of confidence intervals. Moreover, the PC trend was not statistically significant (p-value = 0.334) for the comparison of confidence intervals.

## Discussion

Rheumatic heart disease (RHD) is one of the leading noncommunicable diseases in low- and middle-income countries and accounts for up to 1.4 million deaths annually. There are few contemporary data systematically collected on disease characteristics, treatments, complications, and long-term outcomes in RHD patients.^16^

Despite the magnitude of the problem, Brazil does not have a specific database for this pathology. Thus, because we did not have weekly or monthly data, it was not possible to statistically evaluate disease seasonality. Although these numbers may be underestimated by the lack of a health surveillance strategy during the entire studied period, it is observed that, each year, the number of deaths increases on average 16.94 units, as obtained from the model equation for the trend of the RHD mortality rate (Figure 1C and D). Indeed, ARF and RHD are included in the Brazilian list of preventable death causes for children under 5 years and for the age group of 5 to 75 years. The avoidable or reducible causes of death are defined as those totally or partially preventable by effective health care services, accessible at a certain place and time. Herein, this mortality rate refers to the overall Brazilian population, without distinction of age, with predictive values for 2019 at the magnitude of 8.53 for RHD and 2.68 for ARF, which are higher than the ones from 2017^11^ (6.70 for RHD and 1.94 for ARF), representing an increase of 27.3% and 38.1% for the respective pathologies.

The proposal of the World Health Organization (WHO), to reduce mortality from RHD and other NCDs (noncommunicable diseases) by 25% by the year 2025, requires an understanding of the contemporary characteristics and the use of proven interventions in patients living in endemic countries.^19^ Taking into account our projections, this WHO proposal is far from our reality, which could be associated to the fact that ARF and RHD are diseases of poverty. Moreover, although ARF and RHD have largely disappeared from affluent parts of the world, they remain an important cause of morbidity and mortality in low-income countries and among marginalized sections of society in high-income countries.^20^

These conditions had an impact on the costs of the National Health System, with a remarkable 264% increase in total expenditures with hospitalization for RHD from 1998 to 2016. Considering the current scenario, our predicted values point out the increment of 5.4% for the period from 2017 to 2018 and 1.5% from 2018 to 2019.

The WHO defines secondary prophylaxis as “the continuous administration of specific antibiotics to patients with a previous attack of rheumatic fever, or well-documented rheumatic heart disease. The purpose is to prevent colonization or infection of the upper respiratory tract with GAS and the development of recurrent attacks of rheumatic fever”.^4^ The internationally accepted dose for secondary prophylaxis with BPG in adults is 900 mg (1.2 million IU) intramuscularly. There is some uncertainty regarding the optimal frequency of administration; some studies suggest 2-weekly administration, whereas others report very good outcomes with a 3-weekly regimen^21^ as established by the last Brazilian guideline.^1^

Meanwhile, the value standardized by ANVISA's Drug Market Regulation Chamber for Benzathine Penicillin G is R$ 14.75 or US$ 4.48.^15^ Considering the number of cases due to the evolution of ARF into RHD with its complications (Figure 2) multiplied by the respective costs of procedures (Table 1) we reached the hypothetical value spent in 1 year (R$ 56,726,131.35 or U$ 15,981,534.55; Figure 4). Thus, we highlight that this amount would be enough to carry out secondary prophylaxis of RHD (considered a BPG dose every 3 weeks) in 22,574 people for 10 years. Unfortunately, the low BPG accessibility is not a Brazilian problem, only. Minimal access to BPG was reported in almost all 24 countries in Africa, the Asia-Pacific region, and Central and South America in 2011,^22^ with some respondents indicating no access to BPG at all. Of 39 respondents, 35% indicated that their BPG supply is inadequate to treat all of their patients using the recommended prophylaxis schedules.^22^ Although there are no national data on access to BPG in Brazil, the concern about its lack of availability has increased in recent years.^23^ This lack of an acceptable domestic supply of BPG is a significant problem in several global sites where RF/RHD is prevalent. Without consistent access to an inexpensive and high quality supply of BPG, children in areas with a high prevalence of RF/RHD will remain at risk of developing this crippling and life-threatening condition.^22^

The increasing trend in the RHD and ARF mortality rates, with increments of 27.3% and 38.1% respectively (2017-2019), as well as the comparison between the total costs of the RHD morbidities and the use of BPG, indicates the need for public policies and programs for ARF / RHD control, leading to the early diagnosis and the prevention of disease development and its morbidities. Despite the lack of ARF/RHD control programs in Brazil, this prevention strategy has been already applied in many countries with positive responses, as evidenced by the following data. The 10-year program in Pinar del Rio (Cuba) dramatically reduced morbidity and premature mortality in children and young adults and was cost-effective.^24^ A study carried out in Zambia has shown that understanding public perceptions and behaviors related to neck pain is critical to informing health programs aimed at eliminating new cases of RHD in endemic regions. This cross-sectional study found that pharyngitis is common among school children and adolescents, with women reporting significantly more episodes of sore throats than males. Parents/guardians have varying knowledge of the frequency of sore throats in their offspring, and management of pharyngitis may be suboptimal for many children, with more than one quarter receiving treatment without a qualified evaluation, providing a view of the need for public awareness campaigns aimed at reducing RHD,^25^ which further reinforces the need for greater visibility regarding RHD in Brazil, with program implementation, considering the alarming perspectives of mortality shown in this article.

This increase in mortality may be a matter of discussion considering the possible development of factors, such as better diagnosis, mortality notifications and BPG accessibility. Merely approximately 5% of all carriers of rheumatic fever have a symptomatic acute phase, whereas the majority of patients with severe cardiac rheumatic sequelae are diagnosed only in the final phase of the disease. In fact, these figures may be underestimated, and of these 5% symptomatic individuals, only about 5% need hospitalization,^26^ according to DATASUS data. In Brazil, the PROVAR study^27^ (the country's first large-scale screening program) was implemented in 2014 and revealed an echocardiographic prevalence of 42/1.000 in the preliminary assessment, contrasting with the IBGE prevalence of 7/1.000^1^. This shows that populational screening policies are needed to identify these asymptomatic patients, and it partially explains the increase in prevalence due to better diagnostic methods, but more studies are required to understand the real causes of this increase. The same study shows that although the prevalence of RHD has declined in high-income countries, lack of social and economic development and precarious primary prevention - especially in low- and middle-income countries - perpetuate an environment in which RHD remains endemic and with increasing trends. Moreover, the increased mortality rate is largely due to the stage at which the disease is diagnosed, a classic example being the young woman who discovers severe mitral stenosis only when an acute pulmonary edema is identified during pregnancy.^28^

This progressive increase was also confirmed by another national study,^27^ which justifies the greater availability of echocardiography, with more sensitive criteria, especially for subclinical RHD. In these subclinical cases, echocardiography plays a crucial role because it can establish the diagnosis or even raise suspicion of a possible case in those patients who are going through the last phase of the disease, from the acute manifestations of RFA to the last complications of RHD.^29^

When analyzing our data on mortality from RHD, we see, to a certain extent, the results of non-diagnosed ARF and the cases that were adequately treated in the past. This gap can last 10 to 20 years.^28^ Similarly, by implementing population screening measures to identify the individuals that occupy this gray area, the results will also come after at least a decade.^29^

As in Brazil, the proportion of reports of ARF and RHD in the Pacific islands has increased in recent years, where GAS disease rates seem to be unstoppable.^30^ In the same study, where the annual incidence of ARF was 155 per 100,000, a 41% increase was reported between 2004 and 2009, attributed to improved case detection and reporting of a record and a health program coordinator. However, raising awareness and case reporting is unlikely to account for the high rates of ongoing ARF in this population, as the disease became notifiable in Australia in 1996, an example that should be followed in Brazil, not only notifying hospitalized cases, but compulsorily notifying all cases, allowing greater prophylaxis use. Rates are likely to remain high because of the failure to adequately address socioeconomic determinants of health, increasing the already high rates of infection. Consequently, this remains a significant concern for public health that deserves more attention.

RHD presentation (considering a 10-20 year latency), in the absence of a history of ARF, actually suggests that detection, accurate diagnosis and reporting of ARF remain below ideal. Contributing factors may include lack of training or awareness among health staff, transient health professional staff in remote areas, poor access to medical services, and lack of use of health services due to many factors.^20^

Differences between echocardiographic criteria considerably affect the apparent prevalence of rheumatic heart disease in screening surveys, and emphasize the difficulties in the diagnosis of subclinical disease. Some might argue that there is a wide range of definitions of normality and that echocardiography screening might lead to over- diagnosis. Although controversial, evidence supports a link between mild valvular lesions, detected by echocardiography, and rheumatic heart disease, particularly the substantially higher case detection rates of such lesions in populations at risk for acute rheumatic fever.^31^

Sustained control of rheumatic heart disease at a population level requires a high-functioning health system that meets the needs of vulnerable people. In high-income settings, rheumatic heart disease demonstrates persistent inequality.^32^ For instance, indigenous Australians in the Northern Territory under 35 are 122 times more likely to have rheumatic heart disease than their non-indigenous peers in the same region, reinforcing that a greater focus on RHD prevention and control by strengthening the existing record-based programs (or the development of such programs where they are absent) in countries with high disease burden, improving primary care and raising awareness about ARF and RHD, is critical. Governments, as well as clinicians, should prioritize RHD control to ensure continued funding and recognition of large regional organizations.^30^

In a challenging clinical setting characterized by high ARF/RHD rates, as in Brazil, an Australian study showed a significant improvement in care for people with ARF/RHD in association with the implementation of a continuous improvement quality (CQI) based on participatory research principles. Key findings include improvement in key clinical care indicators, including the administration of scheduled injections of BPG, scheduling injections at the recommended 4-week interval, and periodic review of documentation by a medical specialist, whereas significant improvements in record keeping were also related to ARF/RHD.^33^

Another study carried out in Bangladesh showed that rheumatic fever and rheumatic heart disease are the most common cardiovascular diseases in young people < 25 years of age and are important contributors to cardiovascular morbidity and mortality. It also shows that chronic RHD continues to prevail, and the real burden of disease may be much higher, indicating that large-scale epidemiological and clinical research is needed to formulate evidence-based national policies to address this important public health problem in the future.^34^ As in Brazil, RHD continues to demand a high health and economic rate in African countries, but evidence-based prevention and treatment measures are currently underutilized.^35^

An initial step for Brazil could be based on the report of the African Union Commission (AUC) Social Committee, which described actions that governments must take to eliminate ARF and eradicate RHD: (a) create prospective disease records in sentinel sites (b) decentralization of technical knowledge and technology for the diagnosis and management of ARF and RHD (including echocardiography), (c) establishment of national and regional centers of excellence for cardiac surgery, and (d) promoting international partnerships to mobilize resources and expertise.^36^

Preventive task forces already well established, with the impact of worldwide campaigns, including Brazil, are Pink October and Blue November. We highlight that these two programs are related to prevention of breast cancer (BC) and prostate cancer (PC) mortality, of which magnitude is similar to that of ARF and RHD mortality.

The Blue November began with a movement called *Movember* in Australia in 2003, taking advantage of the celebrations of the World Day to Fight Prostate Cancer, held on November 17, starting its activities in Brazil in 2008. Despite the support of several non-governmental entities, the movement, especially regarding its aspect related to prostate cancer, is repudiated by the Ministry of Health of Brazil and the National Cancer Institute due to the lack of scientific indications for the screening.^37^

Pink October's history dates back to the last decade of the 20^th^ century. In 1997, entities from the cities of Yuba and Lodi in the United States began effectively celebrating and promoting actions aimed at breast cancer prevention, called Pink October. All actions were and are directed towards the prevention and early diagnosis. From 1989-2015 (most recent data available), breast cancer mortality decreased by 39 percent (preventing more than 320,000 deaths).^38^ The first initiative seen in Brazil in relation to the Pink October, was carried out in 2002, and is currently disseminated throughout the country, where there is the involvement of the health teams and the population.^12^

The campaign against rheumatic heart disease needs a strong political will, driven by the awareness and effort of health professionals. The principles that underlie the control of this disease in high-income countries might not apply to developing countries. Where health care finances are very scarce and health is often provided by non-governmental organizations (NGOs), rheumatic heart disease might not be perceived as a priority.^39^ Three successful approaches originating from Central America and the Caribbean, in different economic and political contexts, showed the efficiency of combined strategies consisting of education and primary and secondary prophylaxis.^31^

Some initiatives in this sense have already been taken in Brazil, such as the PROVAR (Rheumatic Valvular Diseases Screening Program) program, being the first large-scale echocardiographic screening program in Brazil, using echocardiography to estimate the prevalence of latent RHD in asymptomatic children between 5 and 18 years of age attending public schools in the underserved areas of cities such as Belo Horizonte, Montes Claros and Bocaiúva, in the Brazilian state of Minas Gerais.^40^

## Conclusion

The analysis of mortality rate trends in Brazil by ARF and RHD are alarming. At the advent of the new millennium, we know little about our real situation due to the lack of a more complete database aimed at this condition. The existing disease load may represent only the tip of the iceberg, since the analyzed data may be underestimated. On a large scale, preferably, national surveys and clinical studies should be conducted to determine the different aspects of RF and RHD in Brazil. The information added by this research would thus help to encourage the real need to formulate national policies to address this public health problem more efficiently in the future. Moreover - why not give a color to rheumatic fever?
